# Anti-Inflammatory, Analgesic and Antioxidant Potential of New (2*S*,3*S*)-2-(4-isopropylbenzyl)-2-methyl-4-nitro-3-phenylbutanals and Their Corresponding Carboxylic Acids through In Vitro, In Silico and In Vivo Studies

**DOI:** 10.3390/molecules27134068

**Published:** 2022-06-24

**Authors:** Fawad Mahmood, Jamshaid Ali Khan, Mater H. Mahnashi, Muhammad Saeed Jan, Muhammad Aamir Javed, Umer Rashid, Abdul Sadiq, Syed Shams ul Hassan, Simona Bungau

**Affiliations:** 1 Department of Pharmacy, University of Peshawar, Peshawar 25120, KP, Pakistan; fawadpharmacist@gmail.com (F.M.); jamshaidkhan@uop.edu.pk (J.A.K.); 2 Department of Pharmaceutical Chemistry, College of Pharmacy, Najran University, Najran 55461, Saudi Arabia; matermaha@gmail.com; 3 Department of Pharmacy, University of Swabi, Swabi 23561, KP, Pakistan; saeedjanpharmacist@gmail.com; 4 Department of Chemistry, COMSATS University Islamabad, Abbottabad Campus, Abbottabad 22060, KP, Pakistan; amirjaved55@gmail.com (M.A.J.); umerrashid@cuiatd.edu.pk (U.R.); 5 Department of Pharmacy, Faculty of Biological Sciences, University of Malakand, Chakdara 18000, KP, Pakistan; 6 Shanghai Key Laboratory for Molecular Engineering of Chiral Drugs, School of Pharmacy, Shanghai Jiao Tong University, Shanghai 200240, China; 7 Department of Natural Product Chemistry, School of Pharmacy, Shanghai Jiao Tong University, Shanghai 200240, China; 8 Department of Pharmacy, Faculty of Medicine and Pharmacy, University of Oradea, 410028 Oradea, Romania

**Keywords:** Michael products, anti-inflammatory, antioxidant, analgesic, carrageenan, COX-2, 5-LOX

## Abstract

In the current study, a series of new (2*S*,3*S*)-2-(4-isopropylbenzyl)-2-methyl-4-nitro-3-phenylbutanals (**FM1-6**) with their corresponding carboxylic acid analogues (**FM7-12**) has been synthesized. Initially, the aldehydic derivatives were isolated in the diastereomeric form, and the structures were confirmed with NMR, MS and elemental analysis. Based on the encouraging results in in vitro COX 1/2, 5-LOX and antioxidant assays, we oxidized the compounds and obtained the pure single (major) diastereomer for activities. Among all the compounds, **FM4**, **FM10** and **FM12** were the leading compounds based on their potent IC_50_ values. The IC_50_ values of compounds **FM4**, **FM10** and **FM12** were 0.74, 0.69 and 0.18 µM, respectively, in COX-2 assay. Similarly, the IC_50_ values of these three compounds were also dominant in COX-1 assay. In 5-LOX assay, the majority of our compounds were potent inhibitors of the enzyme. Based on the potency and safety profiles, **FM10** and **FM12** were subjected to the in vivo experiments. The compounds **FM10** and **FM12** were observed with encouraging results in in vivo analgesic and anti-inflammatory models. The molecular docking studies of the selected compounds show binding interactions in the minimized pocked of the target proteins. It is obvious from the overall results that **FM10** and **FM12** are potent analgesic and anti-inflammatory agents.

## 1. Introduction

Pain and inflammation are closely associated to each other and occur due to complex pathological conditions [1]. Inflammation is basically a response of the cell defense system against tissue injuries or any external stimuli [2]. The onset of inflammation is associated with pain [3]. In the early ages of human development, plants had been used in the management of inflammation and its associated pain [4]. With the development in science and new research, acetylsalicylic acid was first commercialized as an anti-inflammatory drug [5,6]. After the discovery of aspirin, various drugs have been discovered for the management of pain and inflammation, among which NSAIDs (Nonsteroidal Anti-inflammatory Drugs) are the most important and well-known group [7,8]. The pharmacological effects of NSAIDs are due to the inhibition of cyclooxygenase (COX) and lipoxygenase (LOX) enzymes, which are responsible for the metabolism of Arachidonic acid (AA) in the cell membrane and formation of inflammatory mediators such as prostaglandin by COX and leukotrienes by LOX [9]. COX-1 and COX-2 are the two isoforms of cyclooxygenase enzymes that act on the same substrates and catalyze the same reaction but are different in their inhibitor selectivity [10]. COX-1 is mostly involved for maintaining the integrity of the kidney and stomach, while COX-2 produces prostaglandins which mediate pain and inflammation [11,12]. The adverse renal and gastrointestinal effects of NSAIDs are due to COX-1 inhibition, while the inhibition of COX-2 is responsible for the accounts for the therapeutic effects of NSAIDs [13]. In order to prevent such adverse effects of COX-1 inhibition, the scientists turned to design selective COX-2 inhibitors to protect the gastrointestinal tract [14,15].

The oxidation process that takes place in human bodies destroys various cells and tissue and, last, leads to severe illness [16]. It has been observed that the oxidation process may lead to serious conditions such as cancer, various heart diseases and skin problems [17]. Currently, various approaches and techniques are used to eradicate the effect of free radicals [18]. Some of the major sources of antioxidants are natural sources, which may also be helpful in unseen disorders such as stress [19,20]. Day by day, new antioxidants from natural and synthetic sources are improving for the sake of human benefit [21,22]. Most natural products, especially fruits, have specific compounds showing strong antioxidants; however, currently, some of the synthetic compounds also developed have a strong antioxidant capacity [23,24]. Some researchers claims that nitrogenous compounds having a carboxylic acid group show strong antioxidants activities [25].

The Michael reaction of addition nucleophilic moieties to nitro-olefins is a powerful synthetic tool for making the carbon–carbon bond formation [26,27,28]. The reaction has been explored from long ago, and there is time-to-time modification for new outcomes [29]. The organocatalytic Michael addition has been studied from two decades [30]. However, to date, there have been new avenues for the researchers. Modifications or the exploration of new organocatalysts, extending substrate boundaries and sometimes exploring new chemical or biological applications, still is interesting for researchers [31,32,33,34]. The literature shows very limited biological studies on phenylbutanals or their derivatives. In the research early ages, it has been reported as bactericidal [35]. The synthetic derivatives of phenylbutanals have been previously reported with protease inhibitory potentials [36]. This study has been designed to synthesize new Michael products (2*S*,3*S*)-2-(4-isopropylbenzyl)-2-methyl-4-nitro-3-phenylbutanals and their corresponding carboxylic acids for analgesic and anti-inflammatory studies.

## 2. Results

### 2.1. Chemistry of the (2S,3S)-2-(4-isopropylbenzyl)-2-methyl-4-nitro-3-phenylbutanal and Their Carboxylic Acids

**Scheme 1:**
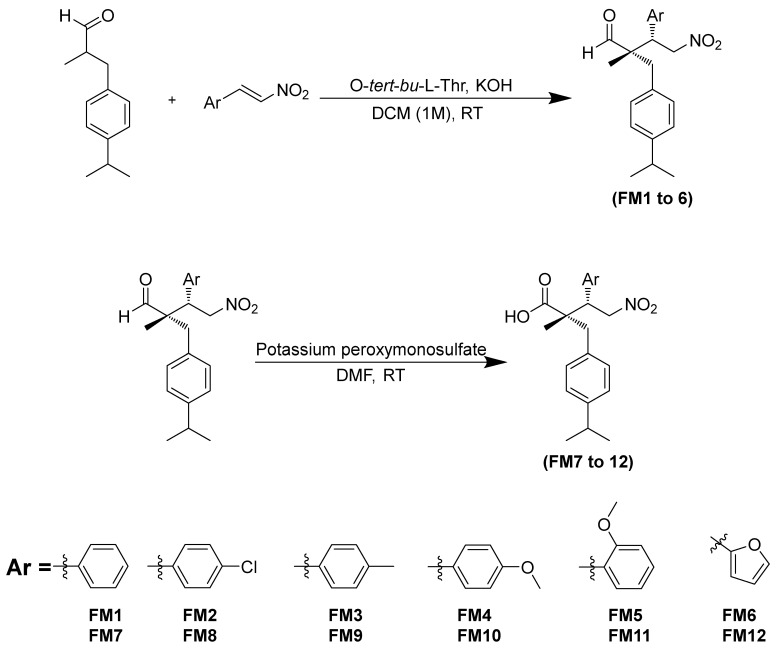
Synthesis of (2*S*,3*S*)-2-(4-isopropylbenzyl)-2-methyl-4-nitro-3-phenylbutanals (**FM1-FM6**) and its corresponding carboxylic acids (**FM7-FM12**).

In the initial synthesis, we have synthesized and purified six nitro-butanal type derivatives having aldehyde functionalities (**FM1-6**). These compounds were purified in the diastereomeric form as the spots on the TLC were not separable. Both the minor and major diastereomers can be seen in the same NMRs. For convenience, we have integrated the whole ^1^H NMR (with both diastereomers) of compounds **FM1-6**. We also performed the preliminary pharmacological activities on these diastereomeric compounds. In the second step reaction, we oxidized the aldehydic derivatives to their corresponding carboxylic acids (**FM7-12**), as shown in Scheme 1. The carboxylic acid derivatives (**FM7-12**) were clearly separable, and only major diastereomers of these compounds were further used in in vitro and in vivo pharmacological assays. The spectra of compounds are provided in the Supplementary Materials.

The individual details of the compounds (**FM1-12**) are given below.

#### 2.1.1. (2*S*,3*S*)-2-(4-isopropylbenzyl)-2-methyl-4-nitro-3-phenylbutanal (FM1)

The compound **FM1** was isolated as a yellowish oil with 83% isolated yield in 24 h reaction time. The observed and calculated retardation factor value (R_f_) was 0.32 in n-hexane and ethyl acetate (4:1). The observed melting point was 149–151 °C. ^1^H NMR (In deuterated chloroform with 400 MHz): 9.65 (s, 1H), 7.37–7.31 (m, 3H), 7.25–7.22 (m, 1H), 7.15–7.10 (m, 3H), 6.91 (d, *J* = 6.62 Hz, 2H), 4.93–4.86 (m, 1H), 4.68 (dd, *J* = 3.85, 13.12 Hz, 1H), 3.85 (dd, *J* = 3.86, 13.17 Hz, 1H), 3.04 (d, *J* = 12.78 Hz, 1H), 2.92–2.81 (m, 1H), 2.41 (d, *J* = 12.73 Hz, 1H), 1.22 (d, *J* = 6.92 Hz, 6H) and 1.06 (s, 3H). ^13^C NMR (In deuterated chloroform with 100 MHz): 206.09, 204.93, 147.79, 135.41, 132.40, 130.34, 130.31, 129.51, 129.34, 129.03, 128.95, 128.92, 128.47, 128.40, 126.71, 126.61, 52.48, 51.98, 49.65, 48.88, 42.13, 40.47, 33.78, 24.05, 17.95 and 16.24. LC-MS: *m*/*z* = 340.42 [M + H]^+^; analysis calculated for C_21_H_25_NO_3_. C, 74.31; H, 7.42; N, 4.13 and O, 14.14. Observed: C, 74.39; H, 7.40 and N, 4.10.

#### 2.1.2. (2*S*,3*S*)-3-(4-chlorophenyl)-2-(4-isopropylbenzyl)-2-methyl-4-nitrobutanal (FM2)

The compound **FM2** was isolated as a clear, oily semisolid with 75% isolated yield in 30 h reaction time. The observed and calculated retardation factor value (R_f_) was 0.35 in n-hexane and ethyl acetate (4:1). The observed melting point was 173–175 °C. ^1^H NMR (In deuterated chloroform with 400 MHz): 9.65 (s, 1H), 7.29–7.07 (m, 6H), 6.94 (d, *J* = 8.25 Hz, 2H), 4.94 (dd, *J* = 11.12, 13.27 Hz, 1H), 4.68 (dd, *J* = 3.65, 13.32 Hz, 1H), 3.84 (dd, *J* = 3.66, 11.13 Hz, 1H), 3.11 (d, *J* = 12.33 Hz, 1H), 2.93–2.81 (m, 1H), 2.75–2.64 (m, 1H), 1.27 (d, *J* = 6.94 Hz, 6H) and 1.14 (s, 3H). ^13^C NMR (In deuterated chloroform with 100 MHz): 206.24, 205.51, 145.65, 145.00, 138.34, 136.98, 132.88, 132.54, 132.04, 131.11, 130.74, 129.86, 129.75, 129.34, 129.17, 129.12, 127.14, 126.85, 126.64, 53.51, 52.14, 49.35, 43.32, 41.31, 37.21, 33.24, 33.00, 24.24, 24.10, 20.17, 18.98, 17.23 and 15.35. LC-MS: *m*/*z* = 374.87 [M + H]^+^; analysis calculated for C_21_H_24_ClNO_3_. C, 67.46; H, 6.47; Cl, 9.48; N, 3.75 and O, 12.84. Observed: C, 67.53; H, 6.45 and N, 3.73.

#### 2.1.3. (2*S*,3*S*)-2-(4-isopropylbenzyl)-2-methyl-4-nitro-3-(p-tolyl)butanal (FM3)

The compound **FM3** was isolated as a white powder with 88% isolated yield in 20 h reaction time. The observed and calculated retardation factor value (R_f_) was 0.38 in n-hexane and ethyl acetate (4:1). The observed melting point was 135–137 °C. ^1^H NMR (in deuterated chloroform with 400 MHz): 9.67 (s, 1H), 7.20–7.12 (m, 6H), 6.93 (d, *J* = 8.11 Hz, 2H), 4.92 (dd, *J* = 11.61, 12.93 Hz, 1H), 4.68 (dd, *J* = 3.81, 13.02 Hz, 1H), 3.84 (dd, *J* = 3.95, 11.53 Hz, 1H), 3.07 (d, *J* = 13.80 Hz, 1H), 2.95–2.84 (m, 1H), 2.77–2.63 (m, 1H), 2.35 (s, 3H), 1.24 (d, *J* = 6.92 Hz, 6H) and 1.07 (s, 3H). ^13^C NMR (In deuterated chloroform with 100 MHz): 206.17, 205.00, 147.69, 147.08, 138.14, 136.24, 132.98, 132.64, 132.24, 130.41, 130.34, 129.67, 129.62, 129.39, 129.21, 129.08, 126.68, 126.66, 126.59, 52.62, 52.15, 49.33, 48.56, 48.31, 42.02, 40.57, 36.35, 33.82, 33.80, 24.17, 24.07, 21.13, 17.77, 16.07 and 13.38. LC-MS: *m*/*z* = 354.45 [M + H]^+^; analysis calculated for C_22_H_27_NO_3_. C, 74.76; H, 7.70; N, 3.96 and O, 13.58. Observed: C, 74.86; H, 7.68 and N, 3.93.

#### 2.1.4. (2*S*,3*S*)-2-(4-isopropylbenzyl)-3-(4-methoxyphenyl)-2-methyl-4-nitrobutanal (FM4)

The compound **FM4** was isolated as a white solid with 78% isolated yield in 24 h reaction time. The observed and calculated retardation factor value (R_f_) was 0.30 in n-hexane and ethyl acetate (4:1). The observed melting point was 117–119 °C. ^1^H NMR (In deuterated chloroform with 400 MHz): 9.64 (s, 1H), 7.34–7.15 (m, 6H), 6.91 (d, *J* = 8.21 Hz, 2H), 4.94 (dd, *J* = 11.04, 12.46 Hz, 1H), 4.72 (dd, *J* = 3.75, 12.52 Hz, 1H), 3.89 (dd, *J* = 3.75, 11.03 Hz, 1H), 3.85 (s, 3H), 3.10 (d, *J* = 11.14 Hz, 1H), 2.91–2.84 (m, 1H), 2.80–2.69 (m, 1H), 1.23 (d, *J* = 6.90 Hz, 6H) and 1.12 (s, 3H). ^13^C NMR (In deuterated chloroform with 100 MHz): 207.14, 206.04, 148.24, 147.54, 139.34, 136.52, 132.99, 132.75, 132.54, 131.87, 130.94, 129.85, 129.74, 129.64, 129.51, 129.38, 127.26, 126.99, 126.45, 58.52, 56.54, 50.34, 49.44, 48.47, 40.54, 40.14, 36.05, 33.52, 32.52, 25.52, 24.99, 21.51, 16.51, 16.15 and 11.41. LC-MS: *m*/*z* = 370.45 [M + H]^+^; analysis calculated for C_22_H_27_NO_4_. C, 71.52; H, 7.37; N, 3.79 and O, 17.32. Observed: C, 71.63; H, 7.35 and N, 3.76.

#### 2.1.5. (2*S*,3*S*)-2-(4-isopropylbenzyl)-3-(2-methoxyphenyl)-2-methyl-4-nitrobutanal (FM5)

The compound **FM5** was isolated as a half white powder with 72% isolated yield in 28 h reaction time. The observed and calculated retardation factor value (R_f_) was 0.34 in n-hexane and ethyl acetate (4:1). The observed melting point was 129–131 °C. ^1^H NMR (In deuterated chloroform with 400 MHz): 9.61 (s, 1H), 7.39–7.26 (m, 4H), 7.18–7.02 (m, 2H) 6.92 (d, *J* = 7.54 Hz, 2H), 4.91 (dd, *J* = 12.51, 13.40 Hz, 1H), 4.80 (dd, *J* = 3.94, 13.42 Hz, 1H), 3.96 (dd, *J* = 3.95, 12.53 Hz, 1H), 3.82 (s, 3H), 3.09 (d, *J* = 11.74 Hz, 1H), 2.90–2.81 (m, 1H), 2.75–2.63 (m, 1H), 1.22 (d, *J* = 6.91 Hz, 6H) and 1.09 (s, 3H). ^13^C NMR (In deuterated chloroform with 100 MHz): 207.51, 206.31, 148.51, 147.30, 140.04, 138.50, 133.04, 132.92, 132.83, 132.64, 131.73, 130.54, 129.99, 129.90, 129.71, 129.58, 128.82, 127.52, 126.79, 57.30, 56.00, 52.74, 49.07, 48.37, 43.53, 41.43, 38.52, 32.89, 32.04, 24.16, 23.74, 20.43, 17.81, 16.63 and 11.74. LC-MS: *m*/*z* = 370.45 [M + H]^+^; analysis calculated for C_22_H_27_NO_4_. C, 71.52; H, 7.37; N, 3.79 and O, 17.32. Observed: C, 71.62; H, 7.35 and N, 3.77.

#### 2.1.6. (2*S*,3*S*)-3-(furan-2-yl)-2-(4-isopropylbenzyl)-2-methyl-4-nitrobutanal (FM6)

The compound **FM6** was isolated a as yellowish semisolid with 95% isolated yield in 20 h reaction time. The observed and calculated retardation factor value (R_f_) was 0.35 in n-hexane and ethyl acetate (4:1). The observed melting point was 161–163 °C. ^1^H NMR (In deuterated chloroform with 400 MHz): 9.59 (s, 1H), 7.40 (dd, *J* = 4.59, 1.84 Hz, 1H), 7.14–7.10 (m, 2H), 7.01–6.93 (m, 2H), 6.34–6.32 (m, 1H), 6.24 (d, *J* = 2.65 Hz, 1H), 4.77 (dd, *J* = 11.31, 12.87 Hz, 1H), 4.57 (dd, *J* = 3.54, 12.88 Hz, 1H), 3.96 (dd, *J* = 3.53, 11.26 Hz, 1H), 2.99–2.94 (m, 1H), 2.89–2.78 (m, 1H), 2.52 (d, *J* = 13.92 Hz, 1H), 1.21 (d, J = 6.91 Hz, 6H) and 1.13 (s, 3H). ^13^C NMR (In deuterated chloroform with 100 MHz): 205.14, 149.64, 147.87, 142.97, 130.40, 130.27, 126.65, 126.59, 110.54, 110.46, 75.32, 74.89, 52.09, 42.03, 41.52, 40.20, 33.73, 30.96, 23.94, 18.08 and 16.56. LC-MS: *m*/*z* = 330.39 [M + H]^+^; analysis calculated for C_19_H_23_NO_4_. C, 69.28; H, 7.04; N, 4.25 and O, 19.43. Observed: C, 69.40; H, 7.02 and N, 4.22.

#### 2.1.7. (2*S*,3*S*)-2-(4-isopropylbenzyl)-2-methyl-4-nitro-3-phenylbutanoic Acid (FM7)

The compound **FM7** was isolated as a yellowish solid with 94% isolated yield. The observed and calculated retardation factor value (R_f_) was 0.18 in n-hexane and ethyl acetate (4:1). The observed melting point was 243–245 °C. ^1^H NMR (In deuterated chloroform with 400 MHz): 12.25 (s, 1H), 7.43–7.37 (m, 3H), 7.35–7.21 (m, 2H), 7.05 (d, *J* = 7.2 Hz, 2H), 6.95 (d, *J* = 6.7 Hz, 2H), 4.96 (dd, *J* = 4.6, 13.8 Hz, 1H), 4.74 (dd, *J* = 3.9, 13.8 Hz, 1H), 3.87 (dd, *J* = 4.6, 3.9 Hz, 1H), 3.07 (d, *J* = 11.2 Hz, 1H), 2.94 (sept, *J* = 6.9 Hz, 1H), 2.54 (d, *J* = 11.2 Hz, 1H), 1.22 (d, *J* = 6.92 Hz, 6H) and 1.06 (s, 3H). ^13^C NMR (In deuterated chloroform with 100 MHz): 177.6, 145.4, 136.2, 132.1, 130.5, 130.0, 129.3, 129.2, 128.9, 128.4, 53.8, 48.4, 42.5, 35.1, 25.3, 15.1 and 14.9. LC-MS: *m*/*z* = 356.42 [M + H]^+^; analysis calculated for C_21_H_25_NO_4_. C, 70.96; H, 7.09; N, 3.94 and O, 18.01. Observed: C, 71.07; H, 7.07 and N, 3.92.

#### 2.1.8. (2*S*,3*S*)-3-(4-chlorophenyl)-2-(4-isopropylbenzyl)-2-methyl-4-nitrobutanal (FM8)

The compound **FM8** was isolated as a half white solid with 95% isolated yield. The observed and calculated retardation factor value (R_f_) was 0.22 in n-hexane and ethyl acetate (4:1). The observed melting point was 259–261 °C. ^1^H NMR (In deuterated chloroform with 400 MHz): 12.21 (s, 1H), 7.30 (d, *J* = 8.1 Hz, 2H), 7.14 (d, *J* = 8.2, 2H), 7.04 (d, *J* = 6.7 Hz, 2H), 6.89 (d, *J* = 6.5 Hz, 2H), 4.94 (dd, *J* = 4.9, 13.4 Hz, 1H), 4.76 (dd, *J* = 5.9, 13.4 Hz, 1H), 3.83 (dd, *J* = 4.9, 5.9 Hz, 1H), 3.05 (d, *J* = 12.0 Hz, 1H), 2.95-2.87 (m, 1H), 2.57 (d, *J* = 11.9 Hz, 1H), 1.27 (d, *J* = 6.87 Hz, 6H) and 1.08 (s, 3H). ^13^C NMR (In deuterated chloroform with 100 MHz): 177.4, 146.1, 137.8, 134.3, 131.4, 130.8, 129.7, 128.2, 128.1, 53.4, 47.9, 41.7, 34.6, 23.7, 16.4 and 13.8. LC-MS: *m*/*z* = 390.87 [M + H]^+^; analysis calculated for C_21_H_24_ClNO_4_. C, 64.69; H, 6.20; Cl, 9.09; N, 3.59 and O, 16.42. Observed: C, 64.78; H, 6.18 and N, 3.56.

#### 2.1.9. (2*S*,3*S*)-2-(4-isopropylbenzyl)-2-methyl-4-nitro-3-(p-tolyl)butanal (FM9)

The compound **FM9** was isolated as a white solid with 90% isolated yield. The observed and calculated retardation factor value (R_f_) was 0.25 in n-hexane and ethyl acetate (4:1). The observed melting point was 233–235 oC. ^1^H NMR (In deuterated chloroform with 400 MHz): 12.23 (s, 1H), 7.24 (d, *J* = 6.5 Hz, 2H), 7.11 (d, J = 6.5, 2H), 7.06 (d, *J* = 5.4 Hz, 2H), 6.81 (d, *J* = 5.5 Hz, 2H), 4.92 (dd, *J* = 3.7, 12.1 Hz, 1H), 4.77 (dd, *J* = 4.6, 12.1 Hz, 1H), 3.85 (dd, *J* = 3.7, 4.6 Hz, 1H), 3.08 (d, *J* = 11.3 Hz, 1H), 2.93–2.85 (m, 1H), 2.55 (d, *J* = 11.0 Hz, 1H), 2.31 (s, 3H), 1.24 (d, *J* = 6.96 Hz, 6H) and 1.05 (s, 3H). ^13^C NMR (In deuterated chloroform with 100 MHz): 175.2, 144.6, 136.0, 135.4, 131.4, 131.8, 130.7, 130.4, 129.1, 128.7, 52.0, 44.8, 42.2, 32.7, 26.3, 24.1, 15.3 and 14.9. LC-MS: *m*/*z* = 370.45 [M + H]^+^; analysis calculated for C_22_H_27_NO_4_. C, 71.52; H, 7.37; N, 3.79 and O, 17.32. Observed: C, 71.63; H, 7.35 and N, 3.76.

#### 2.1.10. (2*S*,3*S*)-2-(4-isopropylbenzyl)-3-(4-methoxyphenyl)-2-methyl-4-nitrobutanal (FM10)

The compound **FM10** was isolated as a white solid with 93% isolated yield. The observed and calculated retardation factor value (R_f_) was 0.20 in n-hexane and ethyl acetate (4:1). The observed melting point was 199–201 °C. ^1^H NMR (In deuterated chloroform with 400 MHz): 12.18 (s, 1H), 7.28 (d, *J* = 8.3 Hz, 2H), 7.15 (d, *J* = 8.3, 2H), 7.09 (d, *J* = 5.7 Hz, 2H), 6.96 (d, *J* = 5.8 Hz, 2H), 4.90 (dd, *J* = 5.2, 13.9 Hz, 1H), 4.82 (dd, *J* = 6.1, 13.9 Hz, 1H), 3.87 (s, 3H), 3.81 (dd, *J* = 5.0, 5.9 Hz, 1H), 3.07 (d, *J* = 11.3 Hz, 1H), 2.90 (sept, *J* = 6.7 Hz, 1H), 2.56 (d, *J* = 12.4 Hz, 1H), 1.24 (d, *J* = 6.91 Hz, 6H) and 1.06 (s, 3H). ^13^C NMR (In deuterated chloroform with 100 MHz): 175.3, 147.6, 140.1, 137.5, 132.6, 132.2, 129.2, 128.8, 128.6, 59.5, 51.0, 49.7, 40.0, 31.4, 21.0, 15.4 and 15.1. LC-MS: *m*/*z* = 386.45 [M + H]^+^; analysis calculated for C_22_H_27_NO_5_. C, 68.55; H, 7.06; N, 3.63 and O, 20.75. Observed: C, 68.68; H, 7.04 and N, 3.61.

#### 2.1.11. (2*S*,3*S*)-2-(4-isopropylbenzyl)-3-(2-methoxyphenyl)-2-methyl-4-nitrobutanal (FM11)

The compound **FM11** was isolated as a white powder with 89% isolated yield. The observed and calculated retardation factor value (R_f_) was 0.19 in n-hexane and ethyl acetate (4:1). The observed melting point was 211–213 °C. ^1^H NMR (In deuterated chloroform with 400 MHz): 12.15 (s, 1H), 7.37–7.28 (m, 3H), 7.16–7.04 (m, 3H), 6.87 (d, *J* = 7.1 Hz, 2H), 4.89 (dd, *J* = 3.8, 12.1 Hz, 1H), 4.87 (dd, *J* = 5.8, 11.9 Hz, 1H), 3.84 (dd, *J* = 3.8, 5.6 Hz, 1H), 3.77 (s, 3H), 3.00 (d, *J* = 11.0 Hz, 1H), 2.87-2.80 (m, 1H), 2.61 (d, *J* = 11.0 Hz, 1H), 1.26 (d, *J* = 6.93 Hz, 6H) and 1.08 (s, 3H). ^13^C NMR (In deuterated chloroform with 100 MHz): 178.2, 145.2, 143.5, 138.3, 133.4, 139.4, 129.0, 129.7, 128.6, 55.1, 50.6, 46.4, 42.4, 32.7, 24.2, 18.4 and 17.1. LC-MS: *m*/*z* = 386.45 [M + H]^+^; analysis calculated for C_22_H_27_NO_5_. C, 68.55; H, 7.06; N, 3.63 and O, 20.75. Observed: C, 68.69; H, 7.04 and N, 3.60.

#### 2.1.12. (2*S*,3*S*)-3-(furan-2-yl)-2-(4-isopropylbenzyl)-2-methyl-4-nitrobutanal (FM12)

The compound **FM12** was isolated as a yellowish powder with 93% isolated yield. The observed and calculated retardation factor value (R_f_) was 0.23 in n-hexane and ethyl acetate (4:1). The observed melting point was 251–253 °C. ^1^H NMR (In deuterated chloroform with 400 MHz): 12.26 (s, 1H), 7.45 (d, *J* = 1.9 Hz, 1H), 7.07 (d, *J* = 5.5 Hz, 1H), 6.91 (d, *J* = 5.5 Hz, 1H), 6.31 (dd, *J* = 1.9, 3.2 Hz, 1H), 6.22 (d, *J* = 3.2 Hz, 1H), 4.78 (dd, *J* = 11.1, 4.87 Hz, 1H), 4.61 (dd, *J* = 4.2, 12.6 Hz, 1H), 3.94 (dd, *J* = 3.9, 11.0 Hz, 1H), 2.98 (d, *J* = 12.9 Hz, 1H), 2.85–2.78 (m, 1H), 2.55 (d, *J* = 13.5 Hz, 1H), 1.21 (d, *J* = 6.6 Hz, 6H) and 1.17 (s, 3H). ^13^C NMR (In deuterated chloroform with 100 MHz): 205.14, 149.64, 147.87, 142.97, 130.40, 130.27, 126.65, 126.59, 110.54, 110.46, 75.32, 74.89, 52.09, 42.03, 41.52, 40.20, 33.73, 30.96, 23.94, 18.08 and 16.56. LC-MS: *m*/*z* = 346.38 [M + H]^+^; analysis calculated for C_19_H_23_NO_5_. C, 66.07; H, 6.71; N, 4.06 and O, 23.16. Observed: C, 66.21; H, 6.69 and N, 4.03.

### 2.2. Antioxidant Results

We tested the antioxidant activities of our compounds (**FM1-12**) using DPPH and ABTS standard methods, and the potencies are summarized in Table 1. We compared our activities with the standard gallic acid, which exhibited IC_50_ values of 09.02 and 03.23 µM against DPPH and ABTS free radicals, respectively. From our results, it can be easily depicted that the oxidized compounds (**FM7-12**) were comparatively potent antioxidants compared to their aldehydic derivatives (**FM1-6**). In aldehydic derivatives, **FM3** and **FM4** were found to be potent in both DPPH and ABTS assays. Similarly, in the oxidized form of compounds (**FM7-12**), compounds **FM10** and **FM12** were found with potent IC_50_ values. The observed IC_50_ values for compounds **FM10** and **FM12** were 08.36 and 15.30 µM in DPPH and 08.90 and 17.22 µM in ABTS assay, respectively. In comparison, the standard gallic acid exhibited IC_50_ values of 09.02 and 03.23 µM against DPPH and ABTS free radicals.

### 2.3. Cyclo and Lipoxygenase Results

The results of in vitro cyclooxygenase and lipoxygenase enzymes’ inhibitions obtained from our synthesized compounds (**FM1-FM12**) are summarized in Table 2. Our compounds were comparatively potent inhibitors of COX-2 enzymes compared to COX-1. In COX-2 results, we observed that the compound **FM4** and its corresponding carboxylic acid (**FM10**) were comparatively more potent, giving IC_50_ values of 0.74 and 0.69 µM, respectively. Both of these compounds have the para-methoxy substitution patterns, which probably have an effect in these specific enzymes’ inhibitions. Similarly, the compound with carboxylic acid and furyl moieties (**FM12**) was the most potent, giving an IC_50_ value of 0.18 µM. On the other hand, the observed results of COX-1 were in a different pattern from that of the COX-2. In comparison, the standard celecoxib exhibited IC_50_ values of 0.042 and 10.87 µM against the COX-2 and -1 enzymes. The calculated selectivity index (SI) was highest for compounds **FM4** (42.8), **FM10** (62.7) and **FM12** (277.1). Though the potency was slightly lower than in standard drugs, however, the SI of our potent compound **FM12** (SI 277.1) was higher than that of standard celecoxib (SI 258.8). A comparatively high SI value shows that the compound would be a good choice specifically in cases of gastric ulcers. The lipoxygenase pathway was also assessed with the available enzyme, and the potencies of our compounds were compared with the zileuton standard drug. Overall, all of our tested compounds were potent inhibitors of 5-lipoxygenase, as can be depicted from the IC_50_ values in Table 2. In 5-LOX assay, five of our compounds were found to be most potent giving IC_50_ values less than one. Compounds **FM2**, **FM4**, **FM7**, **FM8** and **FM12** gave IC_50_ values of 0.64, 0.98, 0.73, 0.87 and 0.43 µM, respectively. The standard zileuton IC_50_ value was 0.50 µM against 5-LOX.

### 2.4. In Vivo Results

Based on the in vitro results, we selected three of our compounds **FM4**, **FM10** and **FM12** for the in vivo studies. In the acute toxicity studies of selected compounds, we only observed very mild seizures and disturbances in breath (temporary) at the highest dose (2000 mg/kg) of compound **FM4**. So, based on this very mild toxicity effect, we excluded the compound **FM4** from in vivo experiments. The other two compounds **FM10** and **12** were found safe even at the maximum dosage. In these two compounds, we observed no behavioral changes in experimental albino mice. A dose of 2000 mg/kg of the compounds was declared safe for animals use. The details of acute toxicity results are summarized in Table 3. According to the organization for economic cooperation and development (OECD) guidelines for the oral acute toxicity, an LD_50_ dose of the >300–2000 was categorized as category 4, and hence the drug was established to be safe.

### 2.5. Carrageenan-Induced Inflammation Results

**Figure 1:**
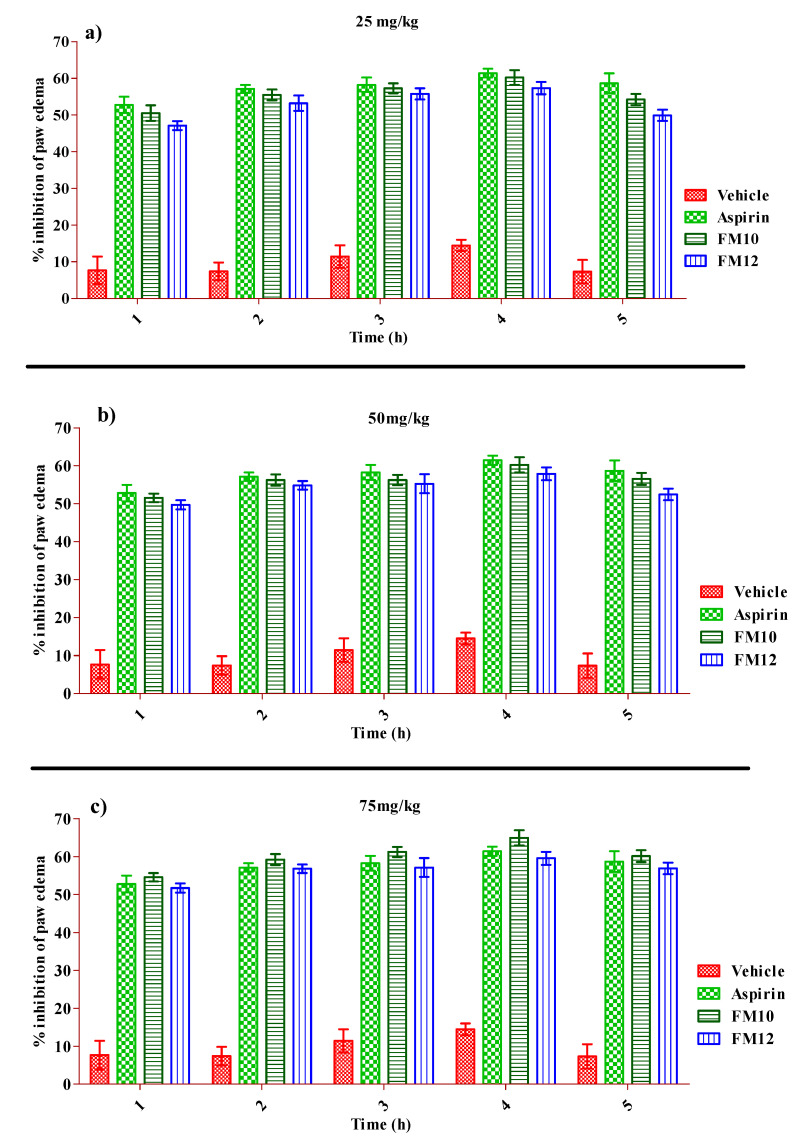
Results of carrageenan assays of compounds **FM10** and **12** at concentrations 25 (**a**), 50 (**b**) and 75 mg/kg (**c**).

Based on the acute toxicity studies, we extended compound **FM10** and **FM12** for in vivo experiments. The carrageenan activity results on concentrations of 25, 50 and 75 mg/kg of the compounds and respective control groups are presented in Figure 1. Overall, our compounds have shown excellent anti-inflammatory activities in this assay. The observed and recorded activity of compound **FM10** was 54.54% at the first hour and remained in observations till the fourth hour. At the fourth hour, the activity was 64.92% at a concentration of 75 mg/kg. The activity profile of our compound was compared with the standard aspirin. The aspirin’s activity was 52.77% at the first hour and 61.43% at the fourth hour of observations. Similarly, the compound **FM12** activity was 51.71 and 59.55% at the first and fourth hours, respectively.

### 2.6. Acetic Acid Induced Analgesic Results

**Figure 2:**
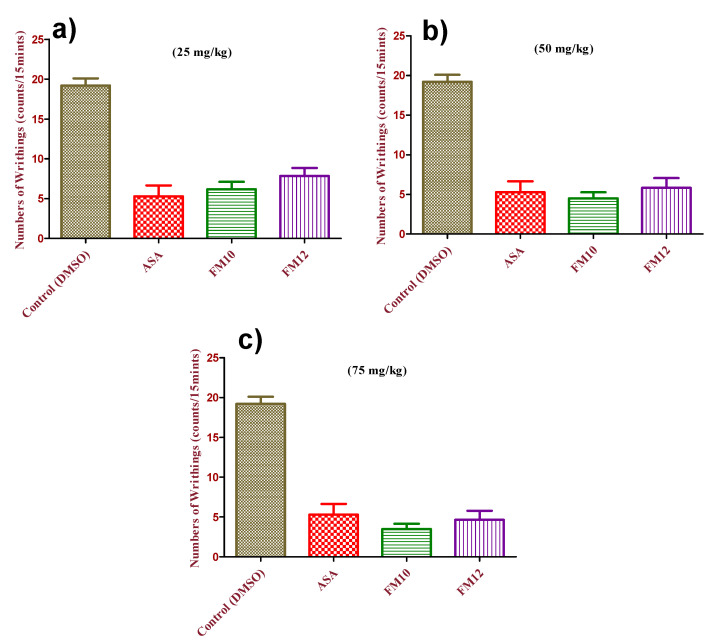
Acetic acid induced test result at different doses of compounds **FM10** and **12**. (**a**) 25 mg/kg, (**b**) 50 mg/kg and (**c**) 75 mg/kg.

A dose dependent analgesic activity profile was observed in the acetic acid induction writhing assay of analgesia. The analgesic potential was indomitable using the acetic acid induction writhing method, which displayed significant potential. Both tested samples were active on the doses of 25, 50 and 75 mg/kg b.wt. The tested compounds **FM10** and **FM12** at the highest doses (75 mg/kg) showed the highest activity when compared to the standard drug (acetyl salicylic acid) (Figure 2c). The standard drug (10 mg/kg) mean inhibition of writhes was 73.01%. **FM10** exhibited a mean inhibition of 85.52% at a high dose (75 mg/kg). Likewise, the compound **FM12** also showed a good inhibition (79.10%) at the same dose, which displayed the highest peripheral analgesic potential. The outcome also pointed out that compounds at a low dose, i.e., 25 as well as 50 mg/kg b.wt, also had moderate to good peripheral analgesic potential, which is displayed in Figure 2a,b.

### 2.7. Results of Formalin In Vivo Assay

The formalin (2%) intraplantar (*i.p*) induction to animals induces a classical biphasic licking response. The time for licking in early phase was 0 to 5 min, which was noted as 57.21 ± 0.42s, and for the late phase (15 to 30 min) it was recorded as 78.07 ± 0.43 s in the control tested group. The pre-treatment of tested compounds at different doses (i.e., 25, 50, 75 mg/kg i.p.) was checked. The compound **FM10** displayed outstanding activity, was significant next to the licking test in both stages, and had an obvious decrease of 87.59% and 76.41% inhibition in the early as well as late phase, as displayed in the Table 4. Likewise, the morphine (5 mg/kg, *i.p.*) injection exhibited clear action in the decrease of both phases of neurogenic pain (88.64% and 93.81%). So, our tested sample, especially compound **FM10,** was close to the standard drug at phase I. Likewise, compound **FM12** in phase I displayed 58.62, 72.78 and 83.54% inhibitions, whilst in phase II it showed 46.94, 60.86 and 72.02% inhibition at various doses such as 25, 50 and 75 mg/kg, correspondingly. Morphine plus naloxone displayed 10.29% potential in the early phase, and in the late phase it exhibited 14.79% activity. The indomethacin with naloxone displayed 10.29% activity in the early phase and 14.79% in the late phase.

### 2.8. Hotplate Analgesic Results

The results of the analgesic potential of the compounds on the hotplate method are summed up in Table 5. The **FM10** was yet again found to display a significant increase in latency time contrast to the standard control (morphine). Primarily, at 15 min, the reaction time means of all three doses of **FM10** were noted as 8.50 ± 0.64, 10.78 ± 0.32 and 13.52 ± 0.65 correspondingly at the doses of 25, 50 and 75 mg/kg b.wt. After sixty (60) minutes, the mean reaction time of the three (3) doses was noted as 6.66 ± 0.33, 8.74 ± 0.46 and 10.36 ± 0.54 min, correspondingly. The initial time reaction at 15 min for the morphine (standard drug) at 5 mg/kg was eminent as 12.88 ±0.26 min, and at 60 min it was noted as 11.22 ± 0.45 min. Likewise, at 15 min, the mean reaction times for compound **FM12** were noted as 7.50 ± 0.64, 9.42 ± 0.74 and 12.44 ± 0.62 min at 25, 50 and 75 mg/kg. Similarly, at 60 min, the reaction times for **FM12** were calculated as 6.45 ± 0.74, 7.39 ± 0.67 and 9.36 ±0.54 min on the similar tested doses.

### 2.9. Molecular Docking Studies

**Figure 3:**
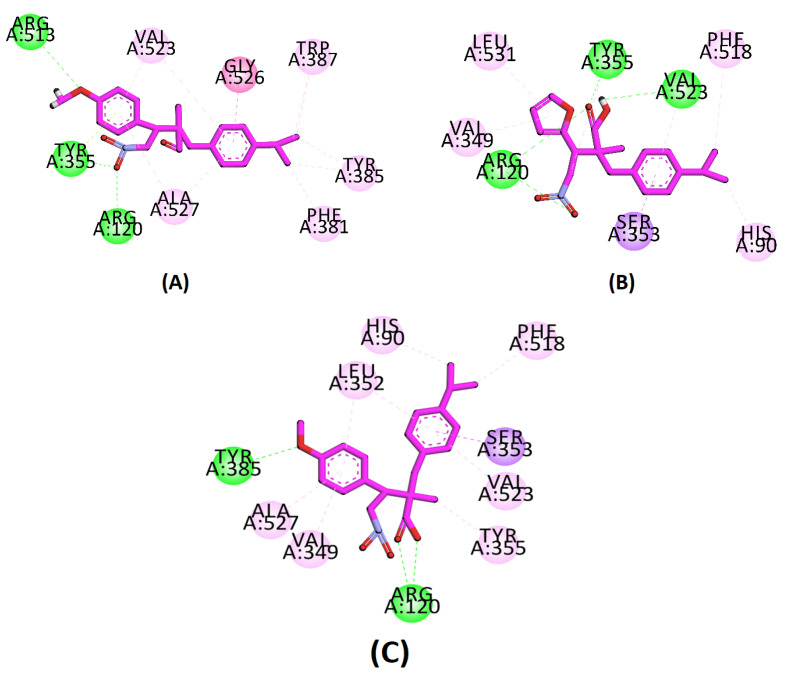
Two-dimensional interaction plots of (**A**) **FM4,** (**B**) **FM12** and (**C**) **FM-10** in active site of COX-2 (PDB ID = 1CX2).

In compound **FM4,** NO_2_ and OCH_3_ moieties form conventional H-bonds with Arg120, Tyr355 and Arg513, while the aromatic ring of anisole forms a π–lone pair interaction with Tyr355 and π–alkyl interaction with Val523 and Ala527. The aromatic part of Cumene shows an amide π-stacked interaction with Gly526 and π–alkyl interaction with Val523 and Ala527, while aliphatic moiety shows a π–alkyl interaction with Phe381, Tyr385 and Trp387 (Figure 3A). In compound **FM12,** NO_2_ and carboxylic acid part form four H-bonds with Arg120, Tyr355 and Val523. The aromatic cumene ring shows a π–sigma and π–alkyl interaction with Ser353 and Val523, respectively, while furan moiety displays π–alkyl interaction with Val349 and Leu531. The compound also exhibits a π–alkyl interaction with His90, Tyr355 and Phe518 (Figure 3B). In compound **FM-10,** carboxylic acid moiety forms two conventional hydrogen bonds with Arg120, while methoxy moiety attached with an aromatic ring also shows a conventional hydrogen bond interaction with Tyr385. One of the aromatic rings shows a π–sigma interaction with Ser353, and FM-12 also shows π–alkyl interactions with His90, Leu352, Ser353, Tyr355, Phe518, Val349, Val523 and Ala527 (Figure 3C).

**Figure 4:**
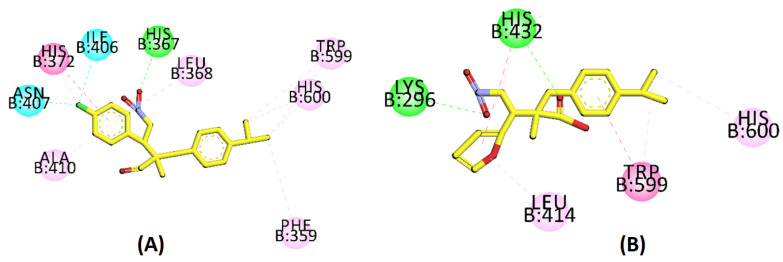
Two-dimensional interaction plots of (**A**) **FM2** and (**B**) **FM12** in active site of 5-LOX (PDB ID = 6N2W).

Compound **FM2** shows a halogen interaction with Ile406 and Asn407 via chlorine moiety, while the aromatic ring of chlorobenzene shows a π–π T-shaped interaction with His372. NO2 moiety form a conventional H-bond with His367, while **FM2** also displays π–alkyl interactions with Phe359, Leu368, His372, Ala410, Trp599 and His600 (Figure 4A). In compound **FM12**, NO2 and carboxylic acid form H-bond interactions with Lys296 and His432; furan and a six membered aromatic ring show π–π stacked and π–π T-shaped interaction with His432 and Trp599, respectively; while **FM12** also shows π–π alkyl interactions with Leu414, His432, Trp599 and His600 (Figure 4B).

**Figure 5:**
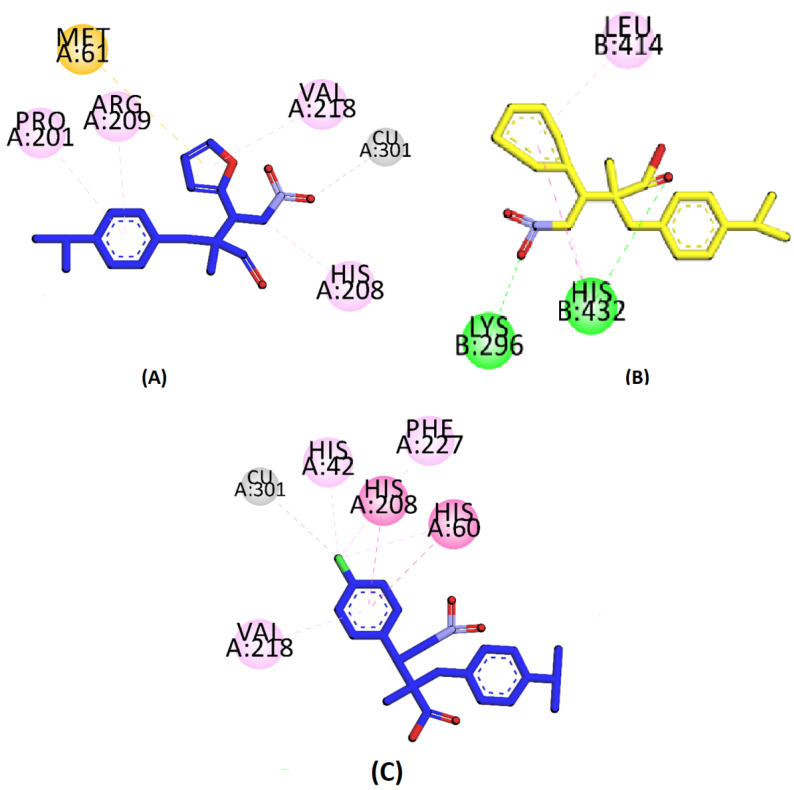
Two-dimensional interaction plots of (**A**) **FM6**, (**B**) **FM7** and (**C**) **FM8** in active site of 6N2W.

In compound **FM-6**, the furan moiety shows a π–sulfur interaction with Met61 and a π–alkyl interaction with Val218. NO2 moiety forms a metal acceptor bond with CU301, while **FM-6** also expresses a π–alkyl interaction with Pro201, His208 and Arg209 (Figure 5A). In compound **FM-7**, the NO_2_ and carbonyl moiety of carboxylic acid form conventional hydrogen bonds with Lys296 and His432, respectively. The benzene ring shows a π–π stacked interaction with His432 and a π–alkyl interaction with Leu414 (Figure 5B). The chlorine moiety of **FM-8** forms a metal acceptor bond with Cu301, and the aromatic ring of chlorobenzene forms a π–π T-shaped and π–π stacked interaction with His60 and His208, respectively. The compound also shows a π–alkyl interaction with His42, Val218 and Phe227 (Figure 5C).

## 3. Discussion

The Michael addition is a powerful tool for synthesizing organic compounds having diverse chemical features [26,32]. The reaction combines a Michael donor and acceptor through C–C bond formation. A variety of Michael donors and acceptors has been studied to synthesized valuable molecules [37]. Enolizable aldehydes, ketones, ketoesters, cyanos and other nucleophilic substance are used as donor molecules. Similarly, nitroolefins, maleimides, vinyl sulfone and other α,β-unsaturated molecules with electron withdrawing groups are used as acceptors [38]. So, by changing any new Michael acceptor or donor, we can synthesize the new compounds. In this research, we reacted 3-(4-isopropylphenyl)-2-methylpropanal with different nitro-olefins to synthesize new compounds. Further, based on the literature, we noticed that the aldehydes are not stable drugs [39]. Therefore, we further oxidized our compounds by converting them into their corresponding carboxylic acids. The literature survey showed that the carboxylic acid-type drugs are potent inhibitors of COX and LOX pathways [9]. The first commercially available drug, aspirin, also has a carboxylic acid functional group.

The cyclooxygenase and lipoxygenase pathways are mainly involved in the inflammation and its associated pain [9,15]. The inhibitors of COX and LOX break up the prostaglandins and leukotrienes production [40]. The prostaglandins and leukotrienes are responsible for inflammation. Therefore, the dual inhibitions of COX and LOX pathways stop inflammation. Among the cyclooxygenases (i.e., COX-1 and COX-2), the selector inhibitors of COX-2 have the advantage of protecting stomach ulceration [41,42]. Therefore, COX-2 selectivity is very important for anti-inflammatory drugs. During our in vitro experiments, we observed that our compounds are selective inhibitors of COX-2. Specifically, by considering our two potent compounds **FM10** and **FM12**, we observed COX-2 selectivity indexes of 62.7 and 277, respectively. In this experiment, the COX-2 selectivity of commercially available standard drug celecoxib was 258.8. In the in vivo experiments, we observed that the carboxylic acid derivatives are comparatively more stable. The unwanted effect associated with aldehydic derivatives might be due to the unstable nature of aldehyde. The aldehyde serves as a pro-drug. Based on our experimental findings, we can claim that we have synthesized new (2*S*,3*S*)-2-(4-isopropylbenzyl)-2-methyl-4-nitro-3-phenylbutanals. Furthermore, we have modified all of our compounds into their respective carboxylic acids for enhance analgesic and anti-inflammatory potentials.

## 4. Materials and Methods

### 4.1. Equipments

The JEOL ECX 400 NMR spectrometer was used. The NMR operated at 400 MHz for proton NMR and 100 MHz for the carbon NMR. The LC-MS used was Agilent Technologies 1200 series (high performance liquid chromatography comprising of a G1315 diode array detector) and ion trap LC-MS G2445D SL. The elemental analyses were conducted with Elemental Vario EI III CHN analyzer. The melting points were determined with Gallenkamp 434.

### 4.2. Synthesis of (2S,3S)-3-aryl-2-(4-isopropylbenzyl)-2-methyl-4-nitrobutanals (FM 1–6)

In a small reaction vessel was added 3-(4-isopropylphenyl)-2-methylpropanal (2.0 mmol, 0.4 μL) in dichloromethane (1 M, 1 mL). To this solution was added further catalytic amounts of O-tertbutyl-L-threonine (0.1 mmol, 17.5 mg) and potassium hydroxide (0.1 mmol, 5.61 mg). The amino acid with KOH was stirred with the aldehyde for 2 to 3 min before adding the Michael acceptor to produce the nucleophilic enamine. Afterwards, the respective Michael acceptor (nitroolefinic compounds in 1.0 mmol) was further added with continued mixing at room temperature. The limiting reagent of the reaction (Michael acceptor) was checked by TLC analysis, and the reaction progress was attributed with the consumption of limiting reagent. At complete conversion (20–30 h), the reaction mixture was quenched with the aqueous portion (10 mL). The organic layer was diluted with dichloromethane (10 mL). The organic layer was separated by a separating funnel. The procedure was repeated three times, and the dichloromethane layers (3 × 10 mL) were combined. Afterwards, anhydrous sodium sulfate was added to it to absorb any moisture. The sodium sulfate was then removed by filtration. The filtrate was washed with dichloromethane to obtain the crude product. The product was concentrated and purified by column chromatography. The structure of compound was confirmed with spectral analysis [26].

### 4.3. Synthesis of (2S,3S)-3-aryl-2-(4-isopropylbenzyl)-2-methyl-4-nitrobutanoic Acids (FM 7–12)

Each of the compounds synthesized in the previous step in one equivalent ratio was diluted in 10 mL DMF-anhydrous and to it was added potassium peroxy-mono-sulfate. The reaction was mixed at 25 °C. When the reaction was completed (3 h), 1 M of hydrochloric acid (HCl) was added to stop it. After workup of the reaction with hydrochloric acid, sodium sulfate anhydrous was added and was filtered. Then, the filtrate was washed out with organic solvent to obtain the crude product [9]. The final product was purified by column chromatography, and the structure was confirmed.

### 4.4. DPPH Free Radical Scavenging Assay

The protocol of Brand-Williams et al. was used for the DPPH assay with some modifications [43]. DPPH (4 mg) was dissolved in methanol (100 mL) to obtain a mixture of 0.01 mM 1,1-diphenyl,2-picrylhydrazyl (DPPH). The stock solution of the various synthesized compounds was prepared in methanol with 1 mg/mL concentration. This stock solution was used to prepare different concentrations of test samples ranging within 1000–62.5 μg/mL. The 0.1 mL of each concentration (1000–62.5 μg/mL) was combined with the DPPH (3 mL) solution in methanol. The solution was kept at 23 °C for 15 min incubation, followed by the absorbance measurement deliberated at 517 nm. Gallic acid was used as a standard drug in this assay. The percentage DPPH radical scavenging potential was measured via the formula [44]:

$$\%\;\mathrm{radical}\;\mathrm{scavenging}\;\mathrm{potential}=\frac{C_{\mathrm{Abs}.}-S_{\mathrm{Abs}.}}{C_{\mathrm{Abs}.}}\times 100$$

where C_Abs._ is the absorbance of the control, and S_Abs_ is the absorbance of test samples/standard.

### 4.5. ABTS Free Radical Scavenging Assay

The total antioxidant activity of test compound (HBH) was estimated using the 2, 2-azinobis [3-ethylbenzthiazoline]-6-sulfonic acid (ABTS). The 100 mL of ABTS solution (7 mM) was added to 100 mL of potassium persulfate (K_2_S_2_O_4_, 2.45 mM) solution, mixed, and kept in the dark for 12 h to generate free radicals. This activated, pre-generated ABTS solution was mixed with different concentrations of the various synthesized compounds (1000–62.5 μg/mL), followed by a suitable dilution with 50% methanol to produce an absorbance of 0.7 at 745 nm. Gallic acid at 2 mg/2 mL of water was used as a standard drug. Likewise, for the test sample, different concentrations (1000–62.5 μg/mL) of the standard drug were made for absorbance measurements at the same wavelength. The 300 μL of each test solution was added to 3 mL of ABTS solution to measure the absorbance at 745 nm through a UV-visible spectrophotometer. A similar volume of each standard solution was taken to determine the absorbance at the same wavelength. The ABTS percent scavenging potential was calculated via the above formula [45].

### 4.6. Cyclooxygenase (COX-1/2) Assay

The COX-1 and 2 enzymes’ inhibitions assays on the synthesized compounds were carried out as per the standard reported method [46]. Initially, the respective enzyme solution was prepared in a concentration of 300 units/mL. The enzyme activation was started with keeping 10 µL of enzyme solution in the cold for up to 10 min. To this enzyme solution was added the substrate solution in HCl (0.1 M Trish buffer with pH of 8.0). The co-factor 50 µL solution contained TMPD (*N*,*N*,*N*,*N*-tetramethyl-*p*-phenylenediamine dihydrochloride, 0.24 mM), hematin (1 mM) and glutathione (0.9 mM). Afterwards, the solutions from synthesized compounds (20 µL in concentration ranging from 31.25 to 1000 µg/mL) and the respective enzyme solution (60 µL) were kept at room temperature for five minutes. The reaction was initiated by adding arachidonic acid (20 µL, 30 mM). The overall solution was incubated for five minutes. Afterwards, the absorbance was recorded on a UV-visible instrument at 570 nm. From the absorbance value of every sample, the percentage inhibition was calculated as per the standard method [47].

### 4.7. 5-Lipoxygenase (5-LOX) Assay

The lipoxygenase inhibition assay on the synthesized compounds was carried out as per the standard reported method [48]. Solutions from synthesized compounds were prepared in concentrations ranging from 31.25 to 1000 µg/mL. The 5-LOX enzyme solution was also prepared at a strength of 10,000 units/mL. The linoleic acid (80 mM) was employed as a substrate in lipoxygenase assay. The buffer (phosphate) was also prepared for the assay having 50 mM strength and a pH of 6.3. The samples of synthesized compounds (250 µL), phosphate buffer (250 µL) and mixture of the enzyme were mixed and incubated for five minutes. Afterwards, the solution of the substrate (0.6 mM, 1000 µL) was mixed with lipoxygenase enzyme mixture with shaking. The absorbance was recorded on a UV-visible instrument at 234 nm. The zileuton was used a control drug in lipoxygenase assay. The percent inhibition was calculated as per the standard method.

### 4.8. Molecular Docking Studies

The molecular docking studies were performed using the MOE software [49,50,51]. Docking studies on the COX-2, 5-LOX and DPPH were carried out to assess binding orientation and ligand–enzyme interactions [9]. All the synthesized compounds were docked into active sites of DPPH, COX-2 and 5-LOX. Protein Data Bank accession codes 5I38, 1CX2 and 6N2W were used to explore crystal structures of DPPH, COX-2 and 5-LOX in complex with Kojic acid, SC-558 and NDGA, respectively. We evaluated docking reliability by re-docking native ligands prior to determining the docking poses of novel compounds. The computed RMSD values (<2.0 Å) were within acceptable ranges.

### 4.9. In Vivo Studies

#### 4.9.1. Experimental Animals

Swiss albino mice of both sexes with an average weight of 30 to 35 g were obtained from the respective section of NIH (National Institute of Health) Islamabad, Pakistan. Written approval was obtained from the Departmental Ethical Committee (No. DREC/20). The animals were reserved in an animal house with the approval of the ethical committee. Throughout the experiments, standard ethical guidelines were followed [52].

#### 4.9.2. Acute Toxicity

Before testing our selected compounds for in vivo experiments, we performed the toxicity test as per the protocol [53]. Four groups of animals were labelled, with eight animals in each group. The control group was given normal saline, while other groups were given different concentrations of the selected compounds. As per the standard protocols, the animals’ behaviors were observed for allergic reactions and mortalities.

#### 4.9.3. Carrageenan-Induced Inflammation

After the acute toxicity studies, the carrageenan-induced inflammation assay was performed on the compounds having a safety profile within limit. Forty (40) albino mice of both sexes were alienated into five different groups, with eight mice in each group. Group I was tagged as the negative control group and was administered dimethylsulfoxide (10 mL/kg, 10% *v*/*v*) and phosphate buffer (150 µL). Group II was tagged as the standard/positive control group and received a dose of aspirin (100 mg/kg in 0.9% normal saline). The remaining groups (III, IV and V) were tagged as experimental groups and received synthesized compounds (25, 50 and 100 mg/kg in DMSO) and Tween-80 in normal saline. After half an hour, carrageenan suspension (0.05 mL, 1% *w*/*v* in saline) was injected into the animals. After the injection of the irritant/carrageenan, the inflammation in the paws was measured by a plethysmometer in intervals (1 to 5 h). The inflammation in the paws of animals in different groups was compared with that of the vehicle, and the percent anti-inflammatory activities were recorded as per the standard method [54].

#### 4.9.4. Acetic Acid Induced Writhing Test

The acetic induced analgesic assay on the compounds **FM10** and **FM12** was performed to determine the role of the peripheral pathway. The albino mice of both sexes were divided into two groups. Compounds **FM10** and **FM12** were administered to both the groups in doses of 25 and 50 mg/kg. After 1 h, acetic acid was injected intraperitoneally in the strength of 10 mL/kg. The negative control was Tween 80 1% solution in the strength of 10 mL/kg. The positive control was diclofenac sodium in the strength of 50 mg/kg intraperitoneally. The activities in animals were determined from the number of stretchings and writings [53].

#### 4.9.5. Formalin-Induced Paw-Licking Test

In this assay, the mice were tagged, and compounds **FM10** and **FM12** were given in concentrations of 25 and 50 mg/kg. Formalin (20 μL, 2.5%) was injected into the animals after 30 min of the compounds. The early phase was initially five minutes, while the late phase was 15-30 min. In both the phases, the mice were under observation for licking. As per the protocols, naloxone (2 mg/kg), indomethacin (10 mg/kg) and morphine (5 mg/kg) were used [28].

#### 4.9.6. Hot Plate Test

The selected compounds (**FM10** and **FM12**) were also tested for anti-nociceptive potentials using a hot plate apparatus. Briefly, test compounds at concentrations of 25 and 50 mg/kg were administered 30 min before observation to the animals and were placed on the surface of hot plate analgesia meter, which was maintained at a temperature of 55 ± 0.2 °C. The response latency, which is a measure of the time taken by animals after the placement of animals on a plate and the licking of paws or jumping, were observed. Morphine (5 mg/kg) was used as a positive. Observations were made after 30, 60 and 90 min of drugs administration [28].

## 5. Conclusions

From our current results, it can be concluded that (2*S*,3*S*)-2-(4-isopropylbenzyl)-2-methyl-4-nitro-3-phenylbutanals (**FM1-6**) and their corresponding carboxylic acids (**FM7-12**) are potential compounds to treat analgesia and inflammation. All of our synthesized compounds (**FM1-12**) are new and were synthesized for the first time. All the compounds are equally potent against the tested in vitro COX-1, COX-2 and 5-LOX targets. The observed IC_50_ values of our most potent compound **FM12** were 0.18, 49.89 and 0.43 μM against COX-1, COX-2 and 5-LOX enzymes. In comparison, the standard celecoxib exhibited IC_50_ values of 0.042 and 10.87 μM (against COX-1 and 2 enzymes), while zileuton gave 0.50 μM against the 5-LOX enzyme. The free radicals within the body can complicate inflammation and the associated pain. Therefore, as a supplementary target, the compounds have also been tested for the in vitro antioxidant assays. We observed that (2*S*,3*S*)-2-(4-isopropylbenzyl)-2-methyl-4-nitro-3-phenylbutanoic acids (**FM7-12**) were comparatively safer in experimental animals. So, based on these observations, we extended potential compounds **FM10** and **FM12** to in vivo studies of analgesia and inflammation. The selected compounds showed a very excellent activity profile in the tested in vivo experiments. We also performed the molecular docking studies of the selected compounds with the target proteins of the respective enzymes. The binding energies showed that our designed compounds are suitable for the COX and LOX targets and can inhibit both of them to treat analgesia and inflammation.

## Figures and Tables

**Table 1:** ABTS and DPPH free radicals scavenging results of compounds **FM1-FM12**.

Samples	DPPH IC_50_ (µM)	ABTS IC_50_ (µM)
FM1	54.35	62.91
FM2	55.36	42.03
FM3	15.08	11.47
FM4	21.08	25.78
FM5	183.73	190.57
FM6	49.70	37.67
FM7	22.54	24.65
FM8	23.50	17.51
FM9	17.02	18.20
FM10	08.36	08.90
FM11	53.32	46.32
FM12	15.30	17.22
Gallic acid	09.02	03.23

**Table 2:** COX-2/1 and 5-LOX inhibitory potentials of the synthesized compounds (**FM1-12**).

Samples	IC_50_ (µM) ± SEM		SI	5LOX IC_50_ (µM) ± SEM
	COX-2	COX-1		
FM1	1.21 ± 0.03	14.76 ± 1.19	12.2	1.81 ± 0.11
FM2	2.51 ± 0.24	38.04 ± 1.65	15.1	0.64 ± 0.01
FM3	3.53 ± 0.18	12.79 ± 1.08	3.6	9.69 ± 0.99
FM4	0.74 ± 0.03	31.70 ± 1.37	42.8	0.98 ± 0.12
FM5	8.15 ± 0.98	58.37 ± 2.08	7.2	16.33 ± 1.02
FM6	4.72 ± 0.08	54.78 ± 1.95	11.6	6.17 ± 0.23
FM7	1.09 ± 0.01	25.31 ± 1.22	23.2	0.73 ± 0.05
FM8	1.99 ± 0.04	61.22 ± 1.84	30.8	0.87 ± 0.08
FM9	8.31 ± 1.01	50.07 ± 1.33	6.0	2.36 ± 0.33
FM10	0.69 ± 0.05	43.29 ± 1.16	62.7	1.77 ± 0.14
FM11	4.25 ± 0.21	35.02 ± 2.13	8.2	11.01 ± 1.14
FM12	0.18 ± 0.01	49.89 ± 1.91	277.1	0.43 ± 0.02
Celecoxib	0.042 ± 0.001	10.87 ± 1.15	258.8	---------
Zileuton	---------	---------	---------	0.50 ± 0.02

**Table 3:** Group of animals and drug quantities given for acute toxicity studies with various synthesized compounds.

Groups	Animals (Male/Female)	Compounds (FM4, FM10, FM12) mg/kg Body Weight
1	5	25
2	5	50
3	5	100
4	5	200
5	5	300
6	5	400
7	5	500
8	5	1000
9	5	2000

**Table 4:** The effects of selected compounds on formalin test.

Samples Names	Dose in mg/kg Body Weight	Time Spent (Licking)			
		0–5 (min.)	Percent (%) Inhibition	15–30 (min.)	Percent (%) Inhibition
Negative control	-	57.21 ± 0.42	-	78.07 ± 0.43	-
FM10	25	18.10 ± 0.59	68.37 ***	37.78 ± 0.73	51.61 ***
	50	12.31 ± 0.47	78.49 ***	25.92 ± 0.98	66.80 ***
	75	7.10 ± 0.92	87.59 ***	18.42 ± 0.56	76.41 ***
FM12	25	23.68 ± 0.68	58.62 ***	41.43 ± 0.92	46.94 ***
	50	15.58 ± 0.48	72.78 ***	30.56 ± 0.65	60.86 ***
	75	09.42 ± 0.57	83.54 ***	21.85 ± 0.87	72.02 ***
Morphine	5	6.50 ± 0.78	88.64 ***	4.83 ± 0.62	93.81 ***
Morphine + Nalaxone	5 + 02	51.32 ± 0.33	10.29 **	66.52 ± 0.40	14.79 ***
Indomethacin + Nalaxone	10 + 02	34.00 ± 0.20	40.57 ***	20.00 ± 0.74	74.38 ***

Data are shown as the mean ± S.E.M; values are significantly variant compared to the control group, and all the data were analyzed via ANOVA followed by Dunnett’s test; *n* = 5, **: *p* < 0.01, ***: *p* < 0.001.

**Table 5:** Results of analgesic activity following hot plate model.

Samples	Dose mg/kg	Reaction Time on Hot Plate in (min)			
		15	30	45	60
-ve control	-	3.91 ± 0.52	4.95 ± 0.39	3.35 ± 0.59	1.73 ± 0.44
FM10	25	8.50 ± 0.64 ***	8.83 ± 0.64 ***	7.52 ± 0.76 ***	6.66 ± 0.33 ***
	50	10.78 ± 0.32 ***	9.26 ± 0.43 ***	9.10 ± 0.57 ***	8.74 ± 0.46 ***
	75	13.52 ± 0.65 ***	12.23 ± 0.44 ***	11.54 ± 0.64 ***	10.36 ± 0.54 ***
FM12	25	7.50 ± 0.64 ***	8.36 ± 0.49 ***	7.27 ± 0.48 ***	6.45 ± 0.74 ***
	50	9.42 ± 0.74 ***	8.52 ± 0.45 ***	8.26 ± 0.47 ***	7.39 ± 0.67 ***
	75	12.44 ± 0.62 ***	11.51 ± 0.62 ***	9.54 ± 0.75 ***	9.36 ± 0.54 ***
Morphine	5	12.88 ± 0.26 ***	12.31 ± 0.62 ***	11.86 ± 0.87 ***	11.22 ± 0.45 ***

The values are existing as the mean ± SEM (*n* = 5). The asterisks display significance level in comparison with negative control: data were analyzed via Dunnett’s test; *** *p* < 0.001.

## Data Availability

Not applicable.

## Supplementary Materials

The following supporting information can be downloaded at: https://www.mdpi.com/article/10.3390/molecules27134068/s1. The supporting information contains a section on chemicals and drugs. Moreover, the following representative spectra of the compounds are provided. Figure S1: ^1^H NMR spectrum of compound **FM1**. Figure S2: ^13^C NMR spectrum of compound **FM1**. Figure S3: ^1^H NMR spectrum of compound **FM3**. Figure S4: ^13^C NMR spectrum of compound **FM3**. Figure S5: ^1^H NMR spectrum of compound **FM6**. Figure S6: ^13^C NMR spectrum of compound **FM6**. Figure S7: ^1^H NMR spectrum of compound **FM12**.
