# Supplementary material for: Anti-Inflammatory, Analgesic and Antioxidant Potential of New (2S,3S)-2-(4-isopropylbenzyl)-2-methyl-4-nitro-3-phenylbutanals and Their Corresponding Carboxylic Acids through In Vitro, In Silico and In Vivo Studies
Source: Molecules. 2022 Jun 24;27(13):4068. doi: 10.3390/molecules27134068 (PMC9268591; doi:10.3390/molecules27134068)
Supplement: Supplementary file 1 [file molecules-27-04068-s001.zip › molecules-1731193-supplementary.pdf]

Supporting Information

Chemical and drugs

Cyclooxygenase (COX-2) was acquired from human recombinant (Catalog No:C0858) and lipooxygenase (5-LOX) from Human Recombinant (Catalog No: 437996), Enzyme substrate including arachidonic acid (CAT No: 150384) and lineolic acid (CAS No: 60-33-3), Indicator and co-factor substances in this study like *N*, *N*_, *N*_-tetramethyl-p-phenylenediamine dihydrochloride (TMPD) CAS No: 637-01-4, glutathione (CAS No. 70-18-8) and hematin (CAS No. 15489-90-4), Carrageenan (CAS No. 9064-67-7), Nalaxon, Arachidonic acid (CAS No. 506-32-1), ABTS (CAS No. 30931-67-0, Sigma Aldrich USA), DPPH (CAS No. 1898-66-4, Sigma Aldrich Chemie GmbH USA), K2S2O4 (Riedel-deHaen Germany), and gallic acid (CAS No. 149-91-7, GmbH USA). these all were purchased from Sigma Aldrich Germany. Buffer solution containing (KH2PO4 & K2HPO4), was of best quality with high purity. Celecoxib, Montelukast was purchased from Pfizer Pharmaceutical and Libra (Pvt.) limited. Tramadol, Diclofenac was purchased from Alliance Pharmaceutical Pakistan.

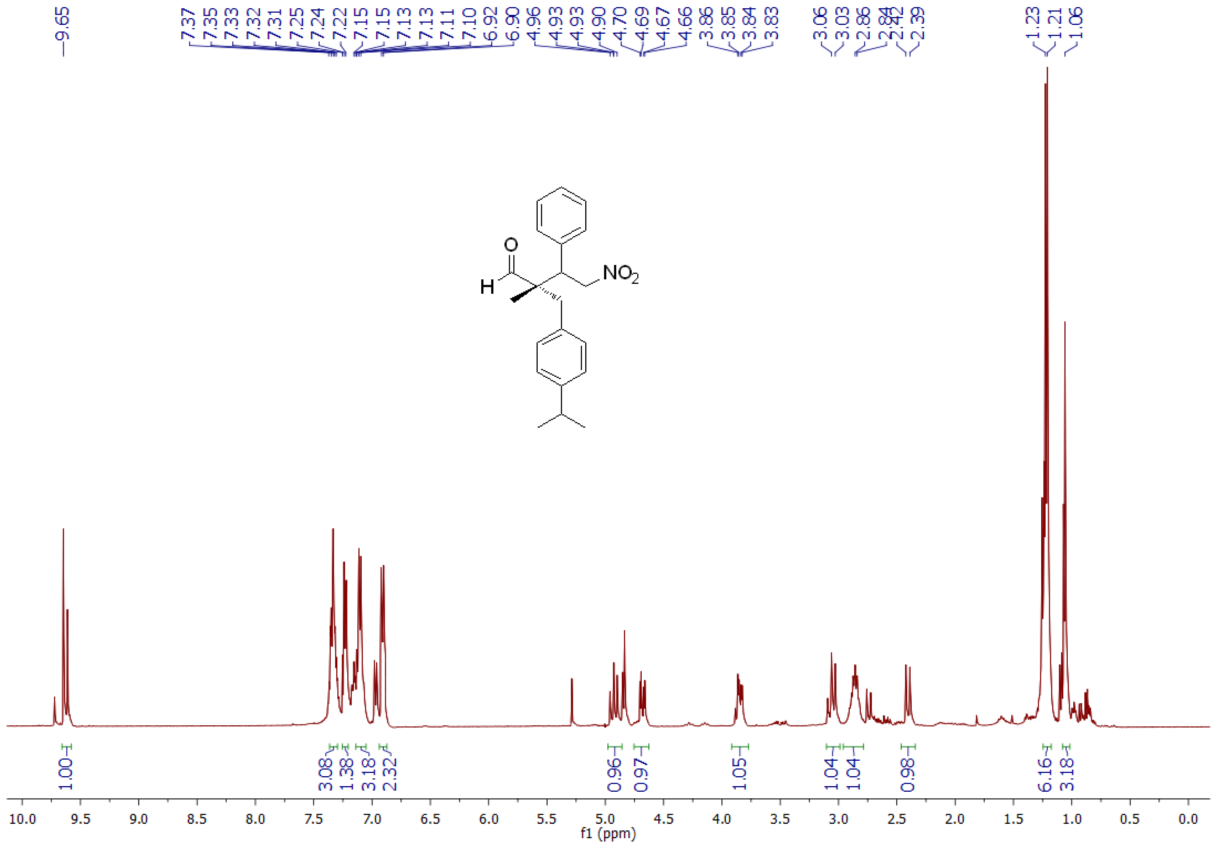

Figure S1. ^{1}H NMR spectrum of compound **FM1**.

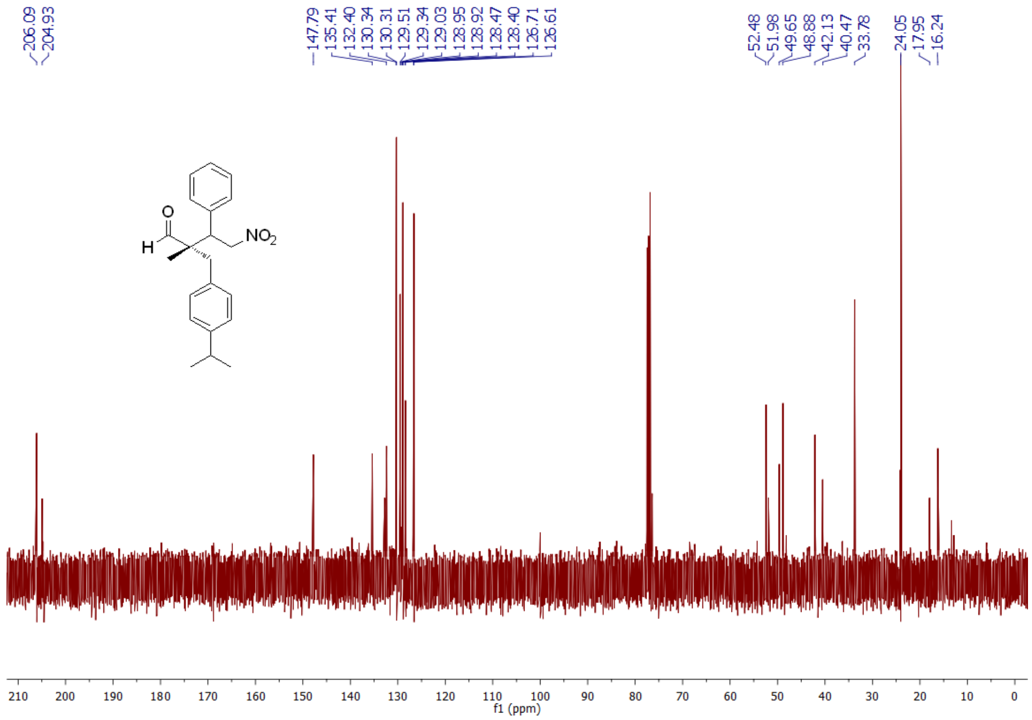

Figure S2. ^{13}C NMR spectrum of compound **FM1**.

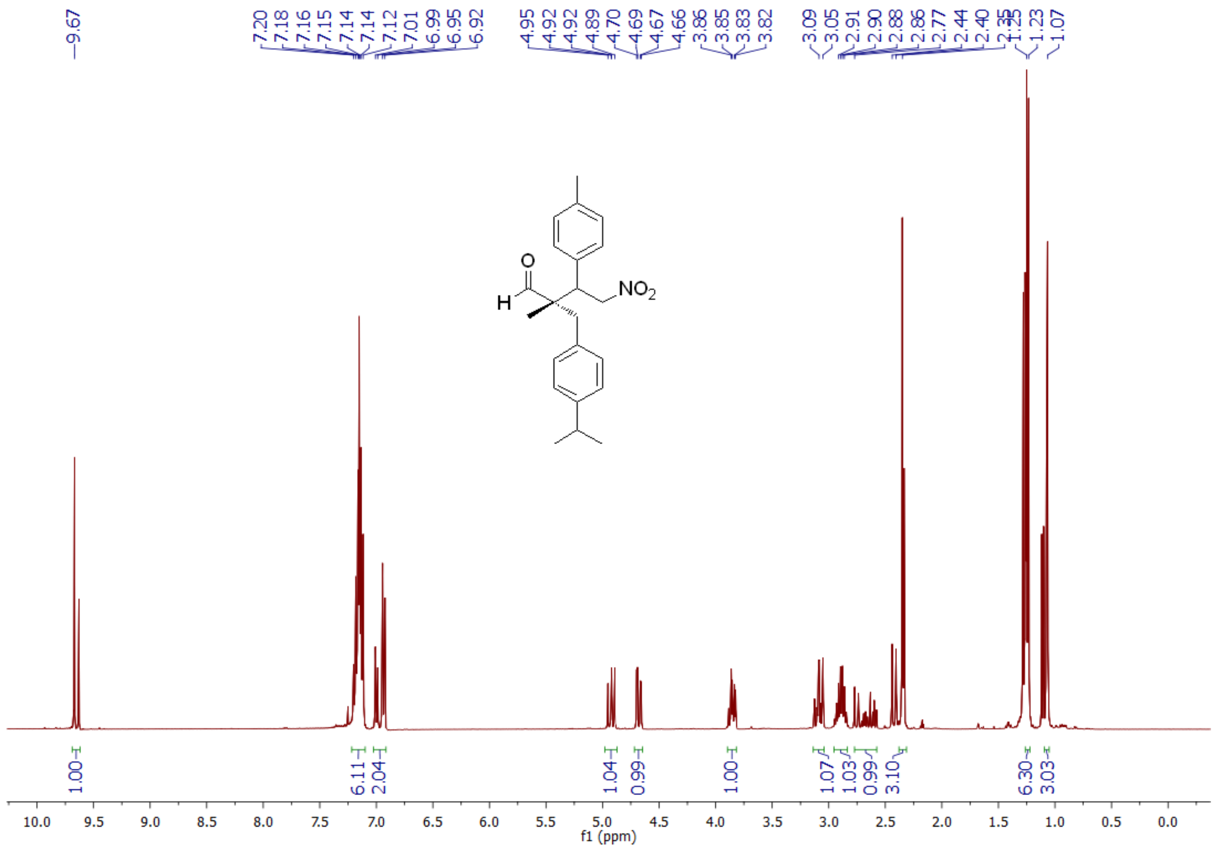

Figure S3. ^{1}H NMR spectrum of compound **FM3**.

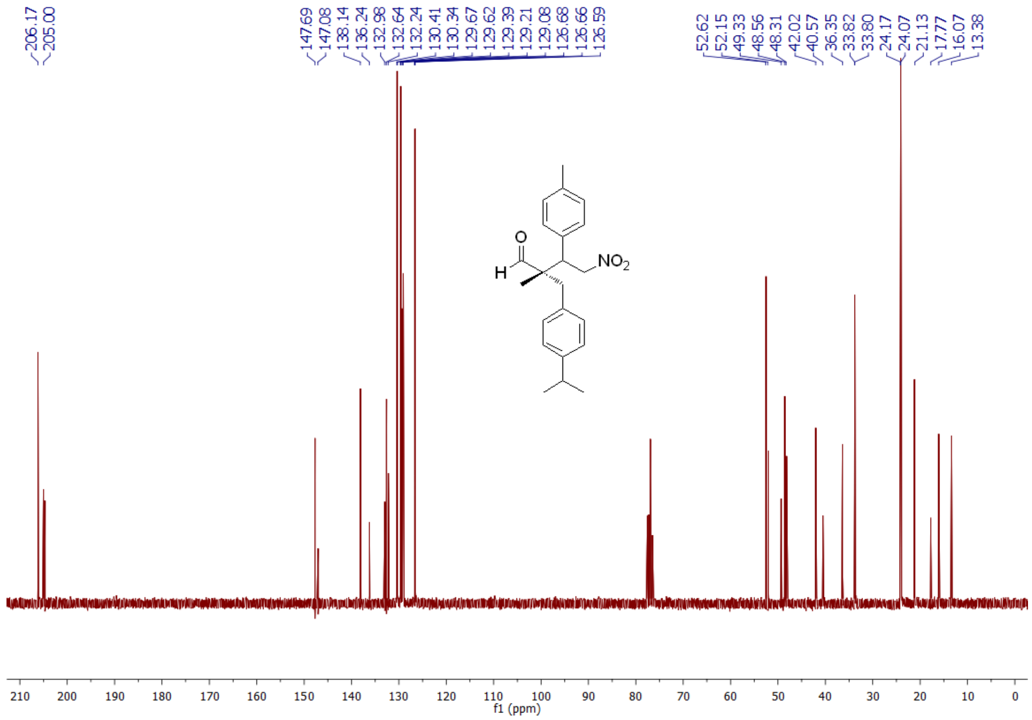

Figure S4. ^{13}C NMR spectrum of compound **FM3**.

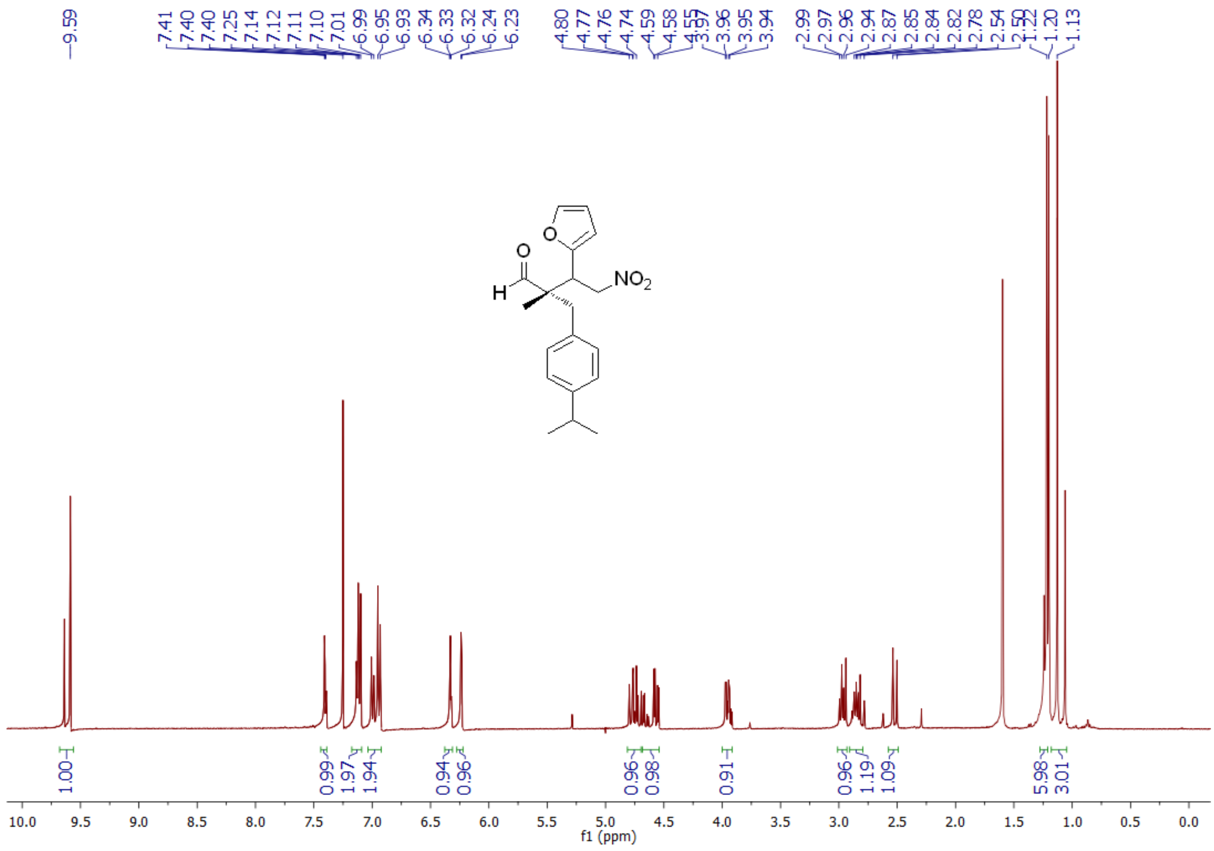

Figure S5. ^{1}H NMR spectrum of compound **FM6**.

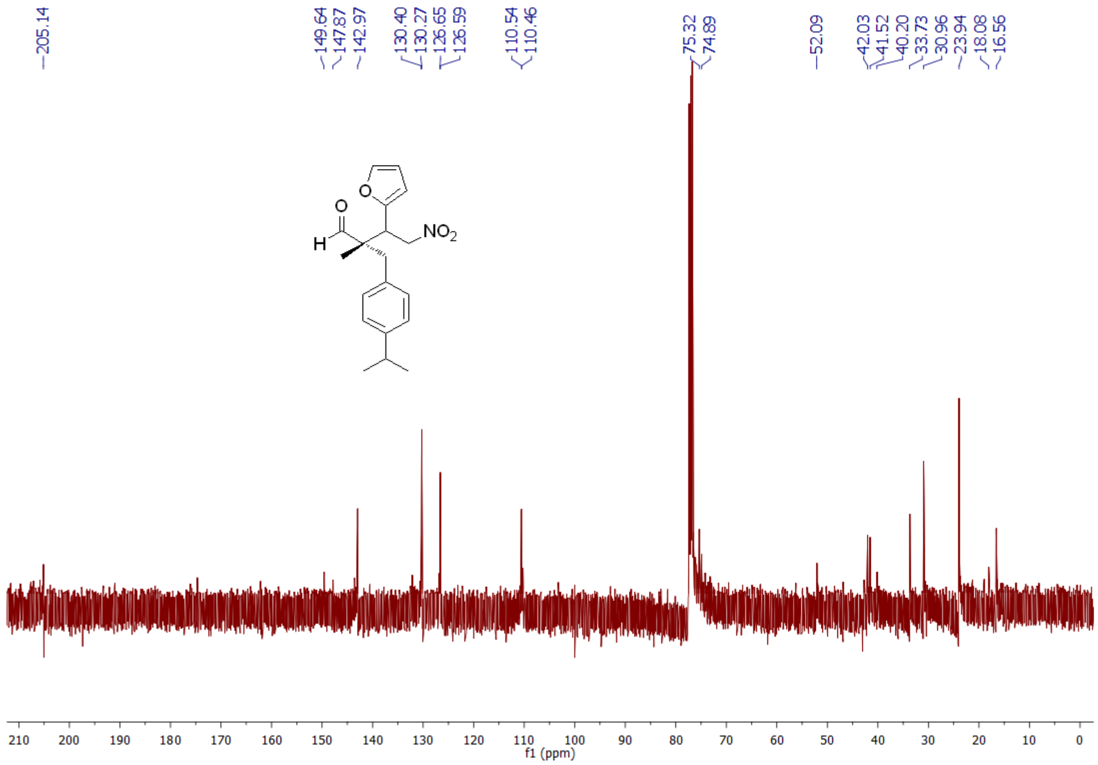

Figure S6. ^{13}C NMR spectrum of compound **FM6**.

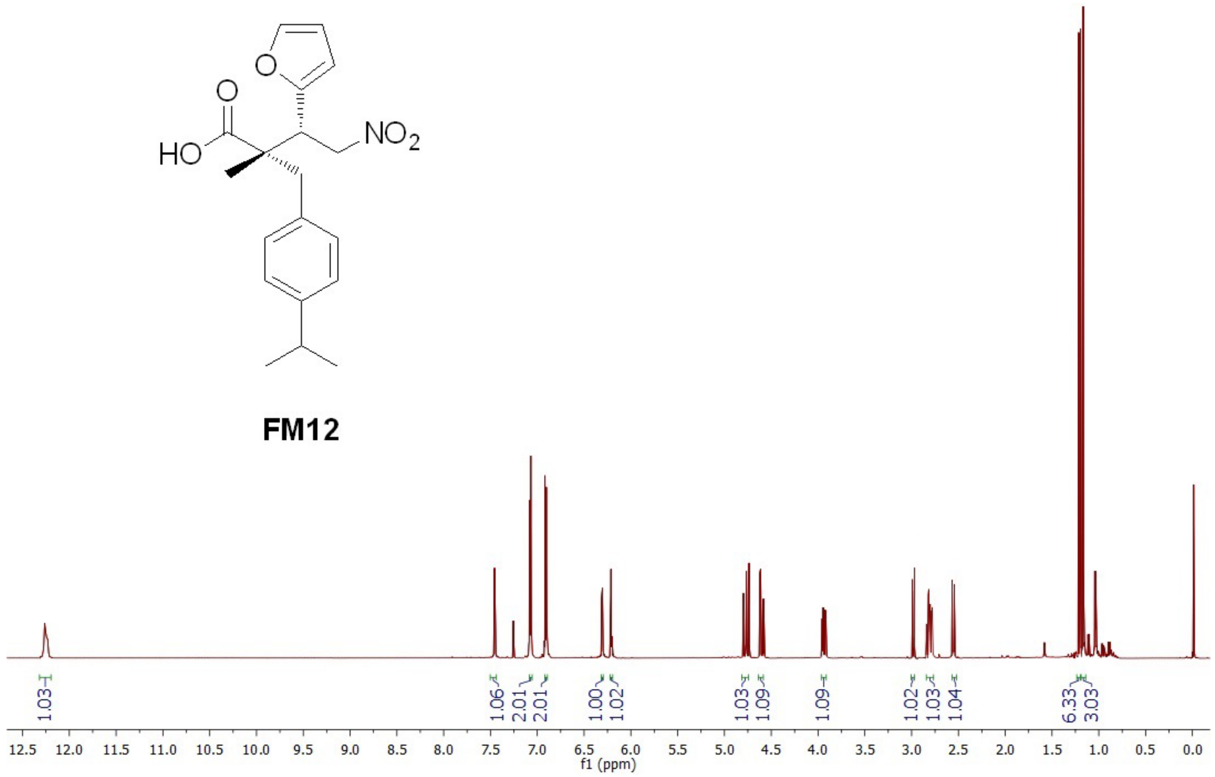

Figure S7. ^{1}H NMR spectrum of compound **FM12**.
